# Supplementary material for: A luciferase prosubstrate and a red bioluminescent calcium indicator for imaging neuronal activity in mice
Source: Nat Commun. 2022 Jul 8;13:3967. doi: 10.1038/s41467-022-31673-x (PMC9270435; doi:10.1038/s41467-022-31673-x)
Supplement: Supplementary file 1 — Supplementary Information [file 41467_2022_31673_MOESM1_ESM.pdf]

SUPPLEMENTARY INFORMATION

A luciferase prosubstrate and a red bioluminescent calcium indicator for imaging neuronal activity in mice

Xiaodong Tian, Yiyu Zhang, Xinyu Li, Ying Xiong, Tianchen Wu, and Hui-wang Ai*

*Corresponding author. Email: huiwang.ai@virginia.edu

Supplementary Figs. 1-18

Supplementary Table 1

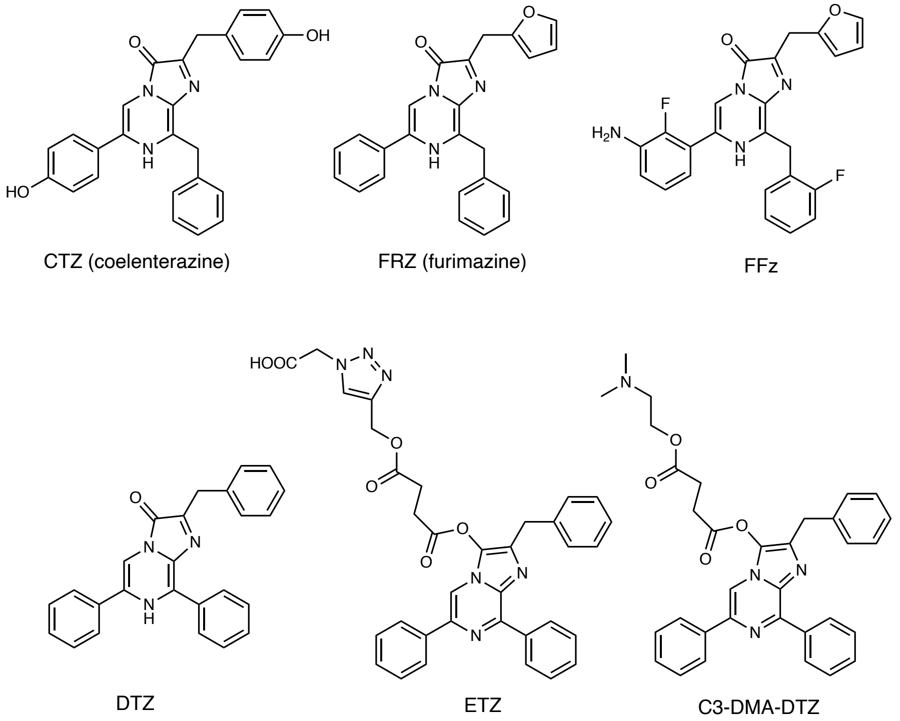

Supplementary Fig 1. Chemical structures of coelenterazine (CTZ) and several representative CTZ analogs.

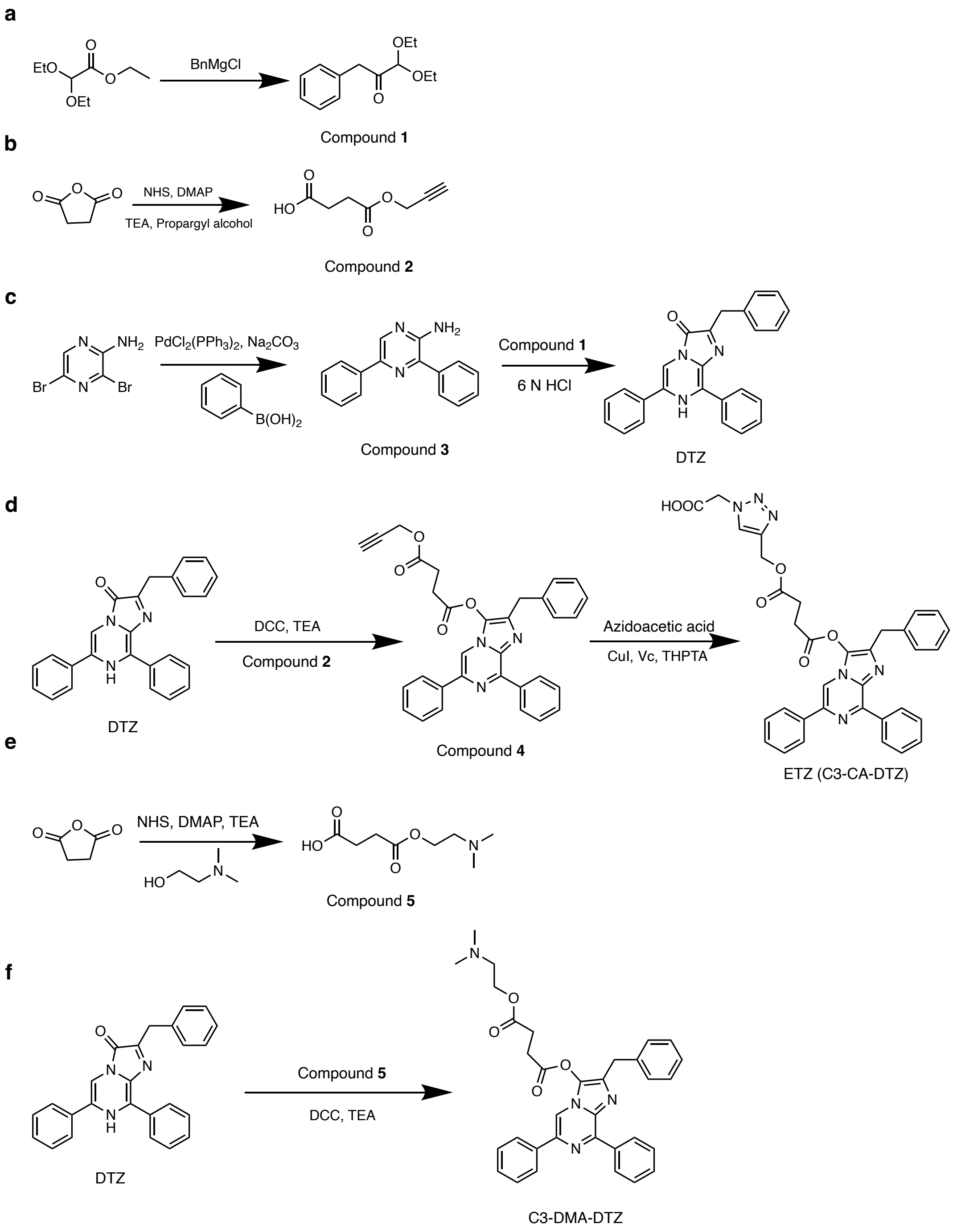

Supplementary Fig 2. Chemical reactions for preparing ETZ and C3-DMA-DTZ. The detailed procedures are described in the Methods section. (**a**) Anhydrous THF, -78 °C 1 h, then RT 1 h, 70%. (**b**) Anhydrous toluene, 110 °C overnight, 65%. (**c**) Step 1: EtOH, reflux overnight, 70%. Step 2: 1, 4-dioxane, 90 °C overnight, 55%. (**d**) Step 1: Anhydrous DCM, RT 30 min, 80%. Step 2: THF and ddH_2O (5:1), RT overnight, 50%. (**e**) Anhydrous toluene, 110 °C overnight, 42%. (**f**) Anhydrous DCM and DMF (10:1), RT 80 min, 15%.

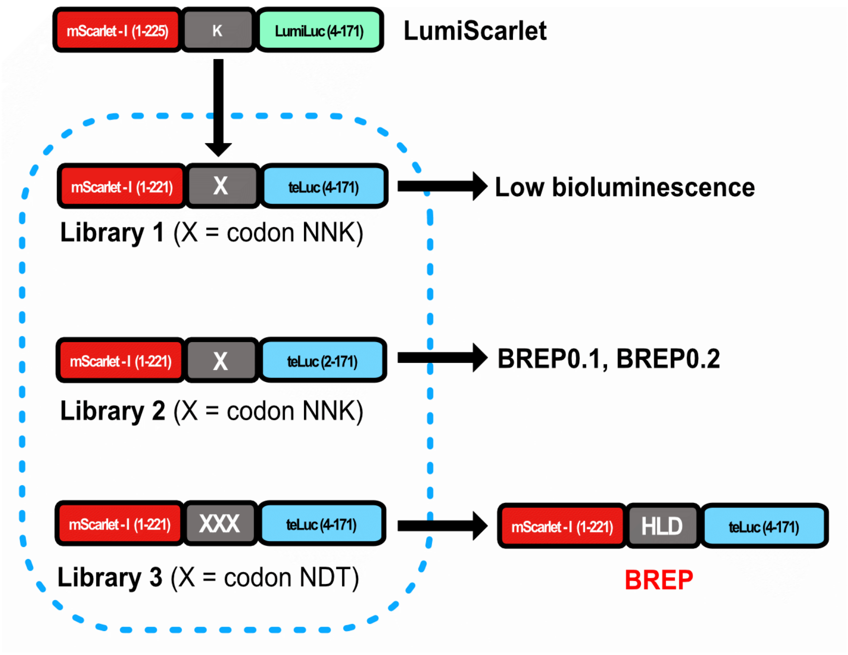

Supplementary Fig 3. Schematic illustration of the workflow to engineer BREP. The numbers in parentheses indicate amino acid residue numbers in the initial proteins. For codons, N refers to A, T, G, or C; K refers to G or T; D refers to A, G, or T. BREP contains a three-residue (HLD) linker and a four-residue truncation of the C-terminus of mScarlet-I. Considering these, mScarlet-I and teLuc in BREP are positioned closer by two residues than mScarlet-I and LumiLuc in LumiScarlet.

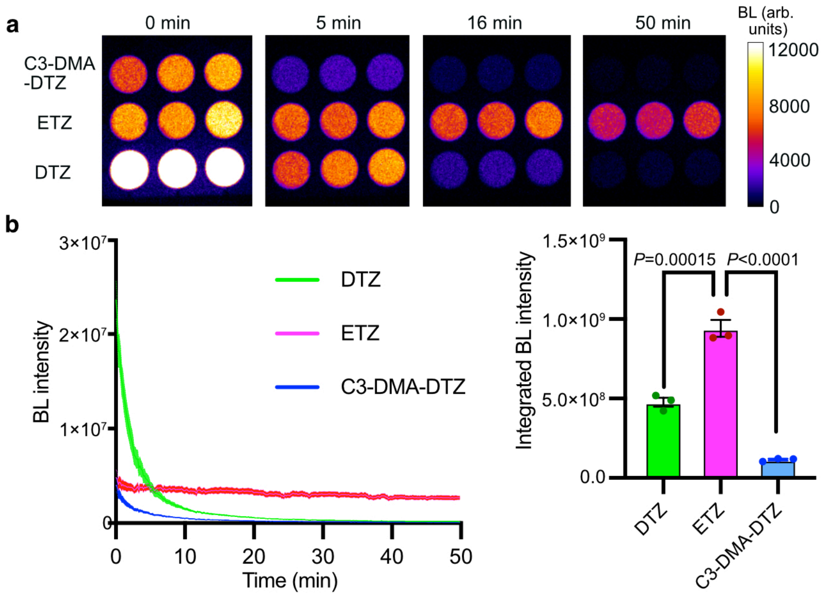

Supplementary Fig 4. Comparison of the synthetic luciferase substrates (DTZ, ETZ, and C3-DMA-DTZ) for bioluminescence in cultured HEK 293T cells. (**a**) BLI of HEK 293T cells transfected with pcDNA3-BREP in wells of a 96-well plate. The substrate concentrations were 25 µM, and images acquired at the indicated time points post substrate additions are presented. (**b**) Left: Bioluminescence intensity over time. Right: Comparison of the integrated bioluminescence intensity (area under the curve) with the residual background subtracted. Data are presented as mean ± s.d. (n=3 cultures). *P* values were derived from ordinary one-way ANOVA followed by Dunnett's multiple comparisons test. The GraphPad Prism software does not provide extract *P* values below 0.0001. Source data are provided as a Source Data file.

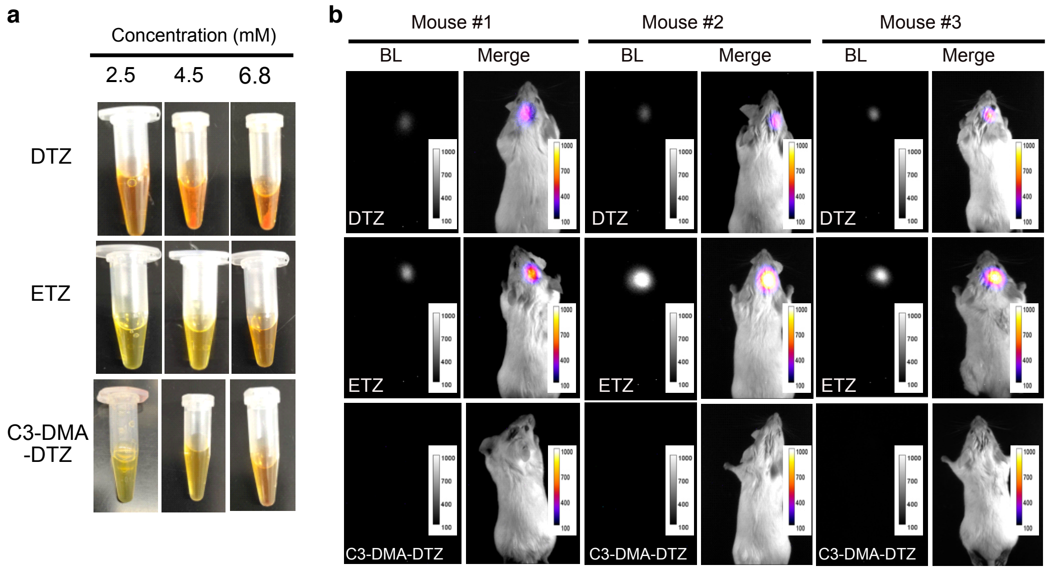

Supplementary Fig 5. Comparison of the synthetic luciferase substrates (DTZ, ETZ, and C3-DMA-DTZ) for imaging HEK 293T cells injected into the brain. (**a**) Solubility tests of the substrates in the intravenous injection buffers, showing the approximate solubility of DTZ, ETZ, and C3-DMA-DTZ to be 2.5, 6.8, or 4.5 mM, respectively. (**b**) BLI of live mice with BREP-expressing HEK 293T cells stereotactically injected into the hippocampus. The substrates were administered via tail vein at their respective, saturation concentrations. Images with peak bioluminescence intensities from each substrate injection were shown in grayscale (left) or pseudocolor overlaid on corresponding brightfield images (right). A group of the images is also presented in Fig. 1d.

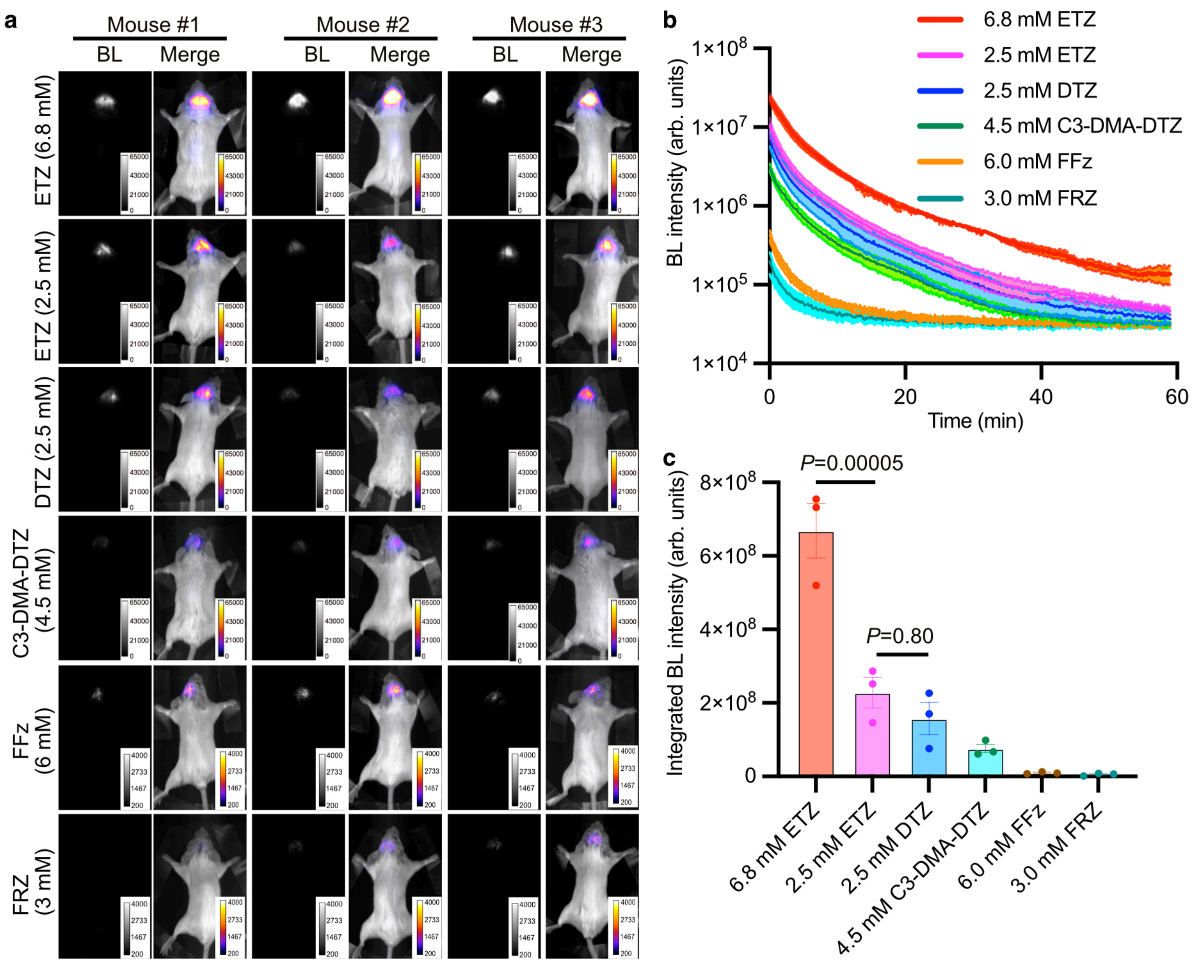

Supplementary Fig 6. Comparison of the luciferase substrates for imaging live mice with BREP AAV-transduced brains. (**a**) BLI of live mice with the hippocampus transduced with BREP AAVs. The substrates (100 µL) at the indicated concentrations were administered via tail vein. Images with peak bioluminescence intensities from each substrate injection were shown in grayscale (left) or pseudocolor overlaid on corresponding brightfield images (right). (**b**) Bioluminescence intensity over time. Results are presented with a logarithmic y-axis. (**c**) Comparison of the integrated bioluminescence intensity (area under the curve) with the residual background subtracted. Data are presented as mean ± s.e.m. (n=3 mice). *P* values were derived from ordinary one-way ANOVA followed by Tukey's multiple comparisons test. Source data are provided as a Source Data file.

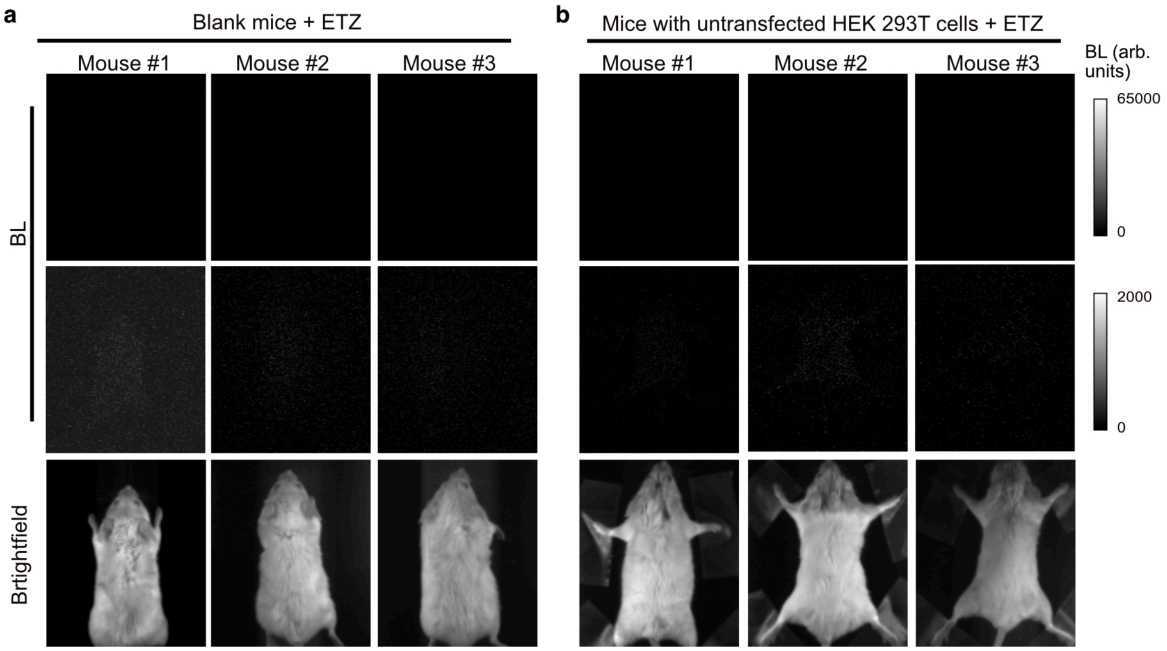

Supplementary Fig 7. Confirmation of very low background bioluminescence from ETZ in mice. (**a**) Images of live mice infused with 100 µL ETZ (6.8 mM) via tail vein. (**b**) Images of live mice with untransfected HEK 293T cells stereotactically injected into the hippocampus and then 100 µL ETZ (6.8 mM) infused via tail vein. Images in the bioluminescence channel are presented using two intensity ranges, and the top row is in the same intensity range as the graphs in Fig. 3b.

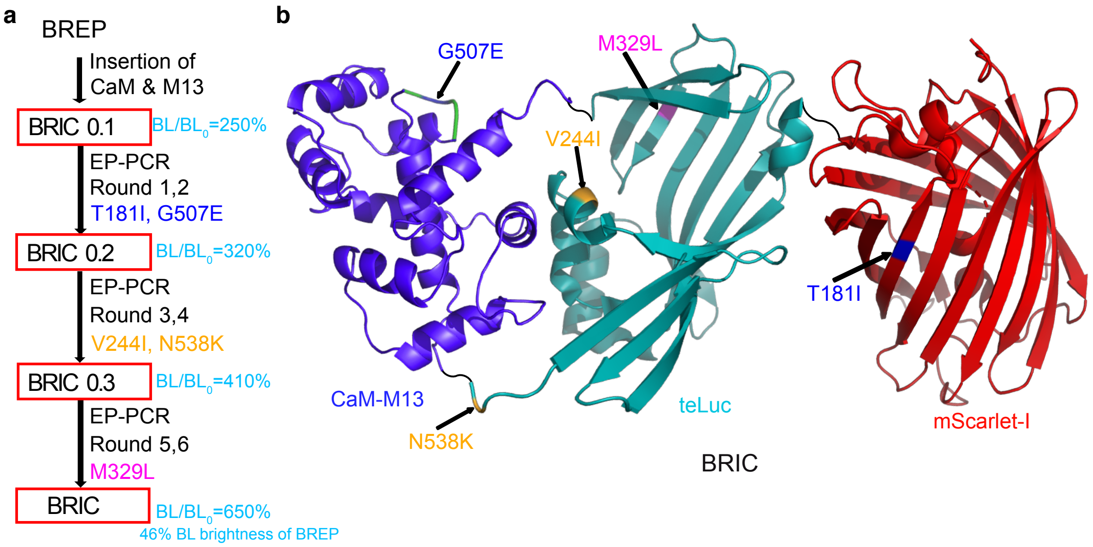

Supplementary Fig 8. Engineering and structural illustration of BRIC. (**a**) Flowchart to show the multistep process to develop and optimize BRIC. Calmodulin (CaM) and M13 were inserted between residues 133 and 134 of teLuc in BREP to create BRIC0.1. Next, six rounds of error-prone (EP)-PCRs were performed to enhance brightness and Ca^{2+}-induced responsiveness, leading to the final BRIC variant with a 6.5-fold (BL/BL_0) turn-on response. BL/BL_0 and mutations gained during the engineering process are also presented. (**b**) Schematic illustration of the domain arrangement of BRIC. Protein Data Bank entries 2BBM, 7MJB, and 5LK4 were used to create this graph. The gained mutations are highlighted.

1–60
BRIC0.1 MVSKGEAVIKEFMRFKVHMEGSMNGHEFEIEGEGEGRPYEGTQTAKLKVTKGGPLPFSWD
BRIC MVSKGEAVIKEFMRFKVHMEGSMNGHEFEIEGEGEGRPYEGTQTAKLKVTKGGPLPFSWD

61–120
BRIC0.1 ILSPQFMYGSRAFIKHPADIPDYYKQSFPEGFKWERVMNFEDGGAVTVTQDTSLEDGTLI
BRIC ILSPQFMYGSRAFIKHPADIPDYYKQSFPEGFKWERVMNFEDGGAVTVTQDTSLEDGTLI

121–180
BRIC0.1 YKVKLRGTNFPPDGPVMQKKTMGWEASTERLYPEDGVLKGDIKMALRLKDGGRYLADFKT
BRIC YKVKLRGTNFPPDGPVMQKKTMGWEASTERLYPEDGVLKGDIKMALRLKDGGRYLADFKT

181–240
BRIC0.1 TYKAKKPVQMPGAYNVDRKLDITSHNEDYTVVEQYERSEGRHLDTLEDFVGDWRQTAGYN
BRIC IYKAKKPVQMPGAYNVDRKLDITSHNEDYTVVEQYERSEGRHLDTLEDFVGDWRQTAGYN

241–300
BRIC0.1 LSQVLEQGGVSSLFQNLGVSVTPIQRIVLSGENGLKIDIHVIIPYEGLSGDQMGQIEKIF
BRIC LSQILEQGGVSSLFQNLGVSVTPIQRIVLSGENGLKIDIHVIIPYEGLSGDQMGQIEKIF

301–360
BRIC0.1 KVVYPVDNHHFKVILHYGTLVIDGVTPNMIDYFGRPYEGIAVFDGKKITVTGTLIMHDQL
BRIC KVVYPVDNHHFKVILHYGTLVIDGVTPNLIDYFGRPYEGIAVFDGKKITVTGTLIMHDQL

361–420
BRIC0.1 TEEQIAEFKEAFSLFDKDGDGTITTKELGTVMRSLGQNPTEAELQDMINEVDADGNGTIY
BRIC TEEQIAEFKEAFSLFDKDGDGTITTKELGTVMRSLGQNPTEAELQDMINEVDADGNGTIY

421–480
BRIC0.1 FPEFLTMMARKMKDTDSEEEIREAFRVFDKDGNGYISAAQLRHVMTNLGEKLTDEEVDEM
BRIC FPEFLTMMARKMKDTDSEEEIREAFRVFDKDGNGYISAAQLRHVMTNLGEKLTDEEVDEM

481–540
BRIC0.1 IREADIDGDGQVNYEEFVQMMTAKGGGSKRRWKKNFIAVSAANRFKKISSSGALELWNGN
BRIC IREADIDGDGQVNYEEFVQMMTAKGGESKRRWKKNFIAVSAANRFKKISSSGALELWKGN

542–575
BRIC0.1 KIIDERLINPDGSLLFRVTINGVTGWRLHERILA
BRIC KIIDERLINPDGSLLFRVTINGVTGWRLHERILA

Supplementary Fig 9. Sequence alignment of BRIC and BRIC0.1. Sequences derived from mScarlet-I, teLuc, calmodulin, and M13 are colored in red, cyan, blue, and orange, respectively. Linker residues are colored in gray. Mutations gained during directed evolution are shaded in yellow.

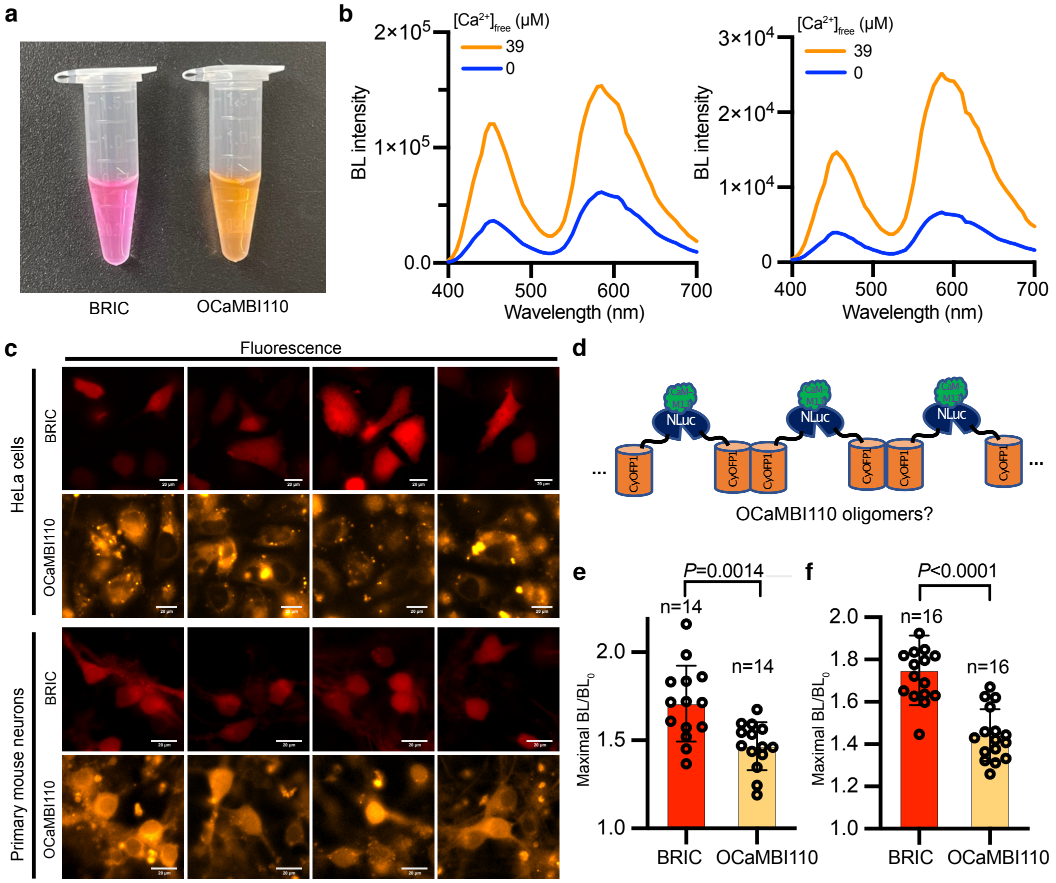

Supplementary Fig 10. ***In vitro*** **characterization of OCaMBI110 and comparison with BRIC in HeLa cells and primary mouse neurons.** (**a**) Photo of the two purified proteins. (**b**) Bioluminescence spectra of OCaMBI110 in the presence of FRZ and the indicated concentrations of free Ca^{2+}. A maximal 2.5-3.7 fold bioluminescence increase (BL/BL_0) was observed using different protein preps. (**c**) Multiple views of BRIC- and OCaMBI110-expressing HeLa cells and primary mouse neurons under the fluorescence channels (Scale bar, 20 µm). Extensive fluorescent puncta were observed in cells overexpressing OCaMBI110 (*e.g.*, HeLa cells 18 h after transfection, and neurons on day 5 after AAV transduction), and these cells with fluorescent puncta showed little bioluminescence activity and were unresponsive to histamine or high K^+. The problem was not seen in BRIC-expressing cells. The experiment was repeated six times with three neuron preparations and similar results were obtained. (**d**) Illustration of the possible formation of oligomers from OCaMBI110, in which CyOFP1 may form intermolecular dimers to bridge individual OCaMBI110 units. (**e**,**f**) Comparison of BRIC and OCaMBI110 for histamine-induced Ca^{2+} in HeLa cells (e) and high K^+-induced depolarization in primary mouse neurons (f). The ratio of the maximal bioluminescence post treatment to the initial intensity is used for comparison. OCaMBI110-expressing HeLa cells were pre-selected to exclude those with fluorescent puncta. In addition, the experiment used neurons on day 4 after AAV transduction, when OCaMBI110 puncta were not obvious. Data are presented as mean ± s.d. with n representing the number of individual cells, and *P* values were derived from unpaired two-tailed *t*-tests. The GraphPad Prism software does not provide extract *P* values below 0.0001. The BRIC data are also used for comparison with BREP in Fig. 2g. Source data are provided as a Source Data file.

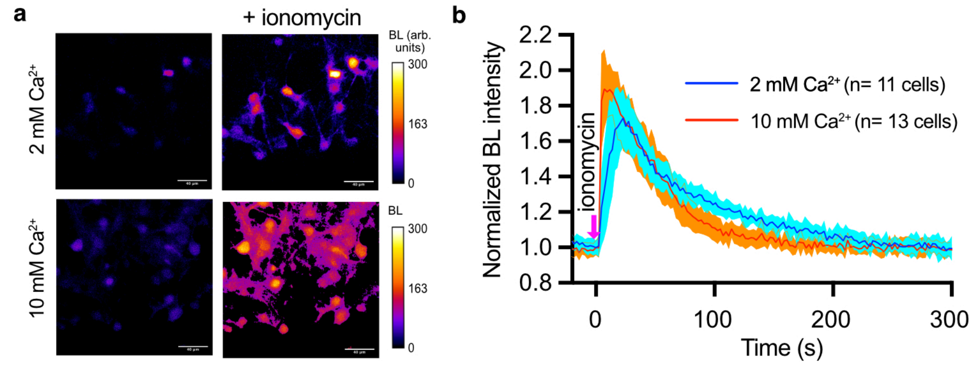

Supplementary Fig 11. Responses of BRIC in primary mouse neurons to ionomycin-induced Ca^{2+} influx. (**a**) Representative bioluminescence images of BRIC-expressing primary mouse neurons in buffers containing 2 mM or 10 mM Ca^{2+} in response to addition of 10 μM ionomycin, a Ca^{2+} ionophore. Scale bar, 40 μm. The experiment was repeated three times and similar results were obtained**.** (**b**) Timecourse of bioluminescence intensity changes. The baselines were corrected using a monoexponential decay model. Data are presented as mean ± s.d.. Source data are provided as a Source Data file.

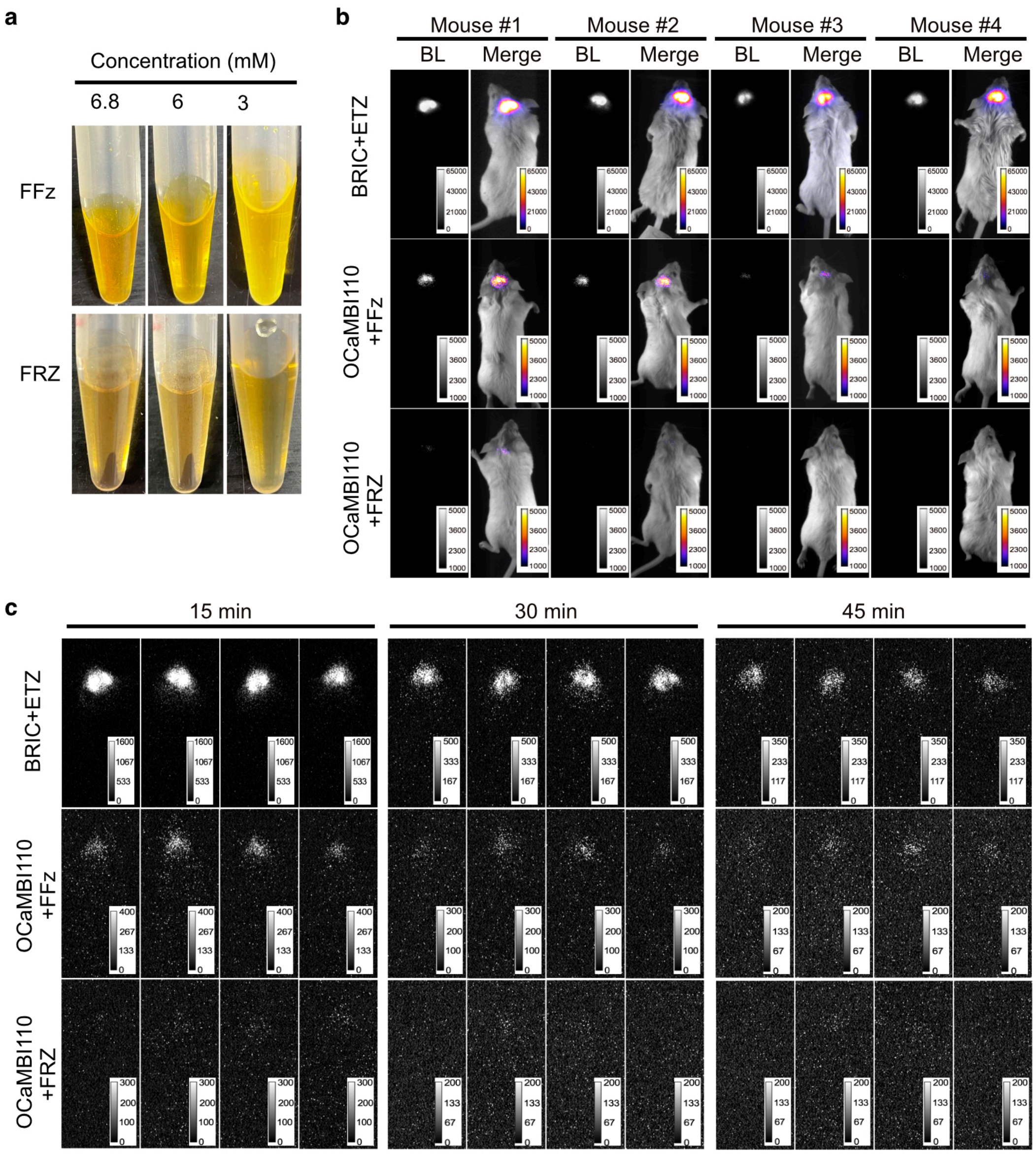

Supplementary Fig 12. Brightness comparison of BRIC and OCaMBI110 in the hippocampus in live mice. (**a**) Solubility tests of FFz and FRZ in the intravenous injection buffers, showing their approximate solubility to be 6 and 3 mM, respectively. (**b**) Bioluminescence images of live mice with the hippocampus transduced with BRIC or OCaMBI110 AAVs. Mice in the BRIC group were administered 6.8 mM ETZ via tail vein. Mice in the OCaMBI110 groups were administered 6 mM FFz or 3 mM FRZ via tail vein. Images at the beginning of individual imaging sessions (right after substrate injection) were shown in grayscale (left) or pseudocolor overlaid on corresponding brightfield images (right). A group of the images is also presented in Fig. 3. (**c**) Bioluminescence images in grayscale at 15, 30, and 45 min post substrate injections. In panels b and c, results from different experimental groups are presented with varying intensity ranges due to large intensity differences.

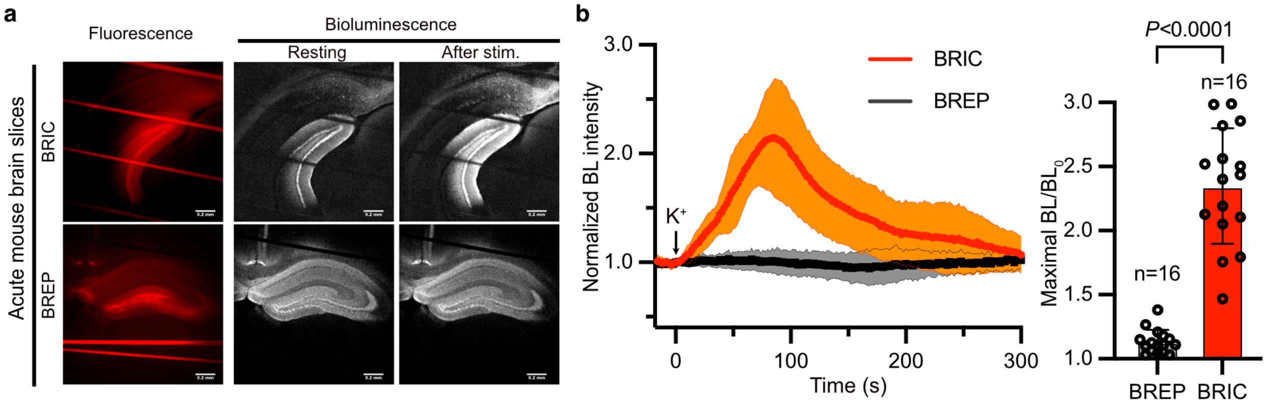

Supplementary Fig 13. Bioluminescence imaging of high-K^+-induced Ca^{2+} in acute mouse hippocampal slices. (**a**) Reprehensive fluorescence and bioluminescence images of brain slices expressing BRIC or BREP (Scale bar, 0.2 mm). A peristaltic pump was used to introduce a high KCl buffer via a 42-s period. The final K^+ concentration was 30 mM. (**b**) Quantification of bioluminescence intensities of brain slices in response to high K^+. The baselines were corrected using a monoexponential decay model. Data are presented as mean ± s.d., n=16 randomly chosen areas from 5 slices. *P* values were derived from unpaired two-tailed *t*-tests. The GraphPad Prism software does not provide extract *P* values below 0.0001. Source data are provided as a Source Data file.

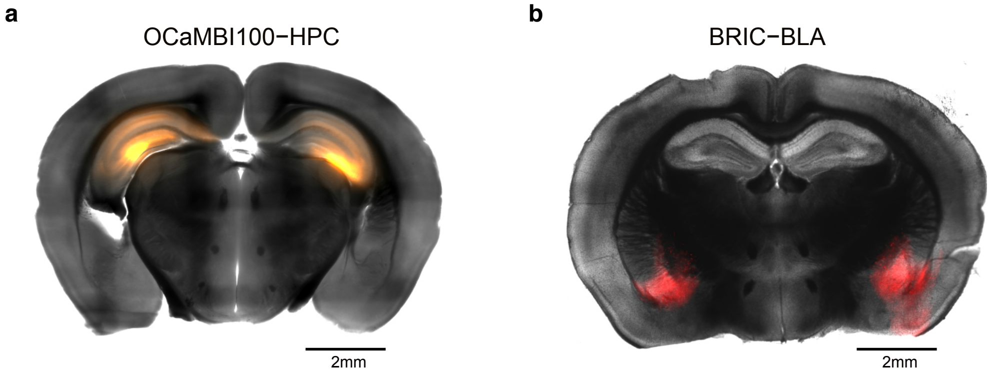

Supplementary Fig 14. Image of acute brain slices prepared from AAV-transduced mice. (**a**) An acute brain slice prepared from a mouse with OCaMBI110 virus injected into the hippocampus (HPC). (**b**) An acute brain slice prepared from a mouse with BRIC virus injected into the basolateral amygdala (BLA). The fluorescence channel (orange or red) is overlaid on the corresponding grayscale brightfield image. Scale bar, 2 mm.

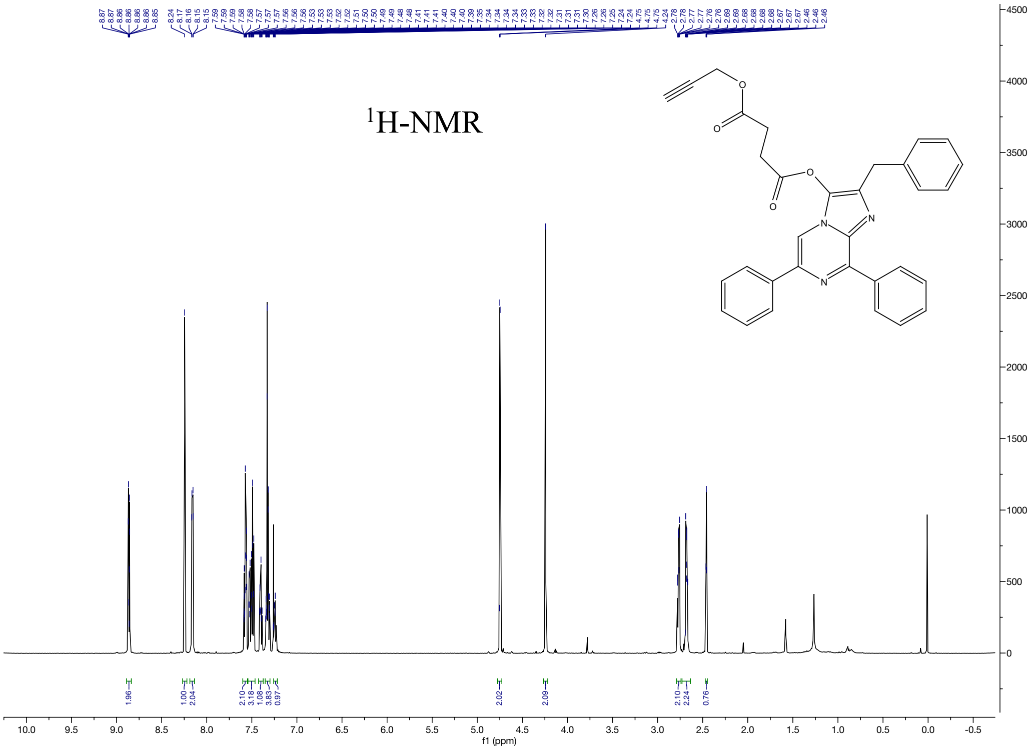

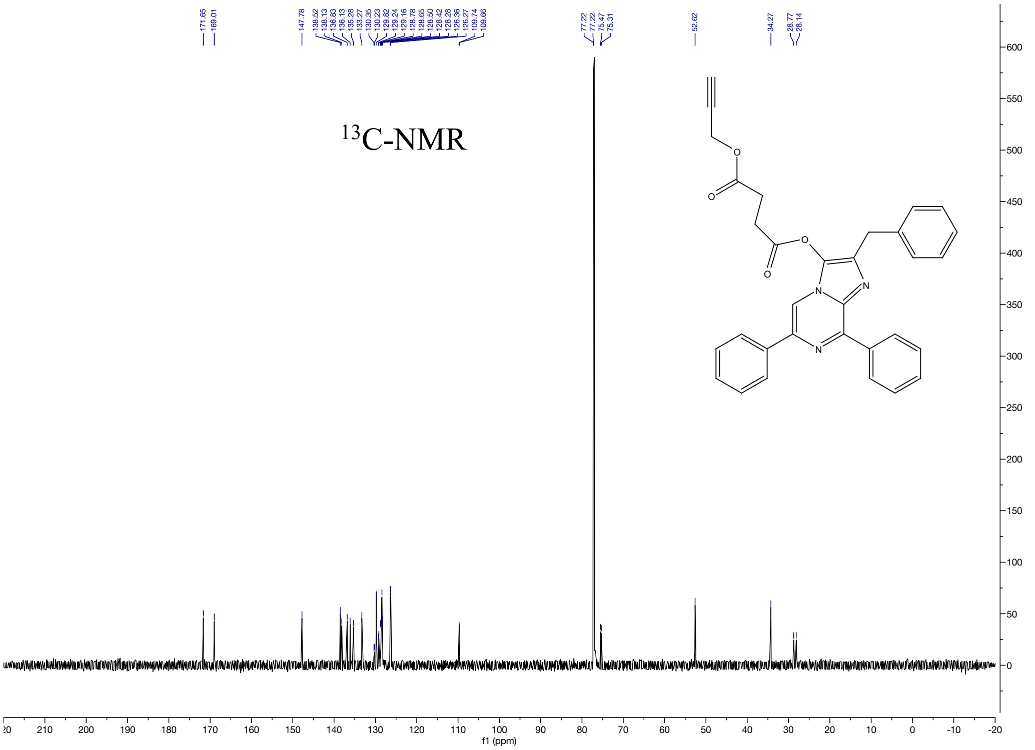

Supplementary Fig 15. NMR spectra for the indicated compound.

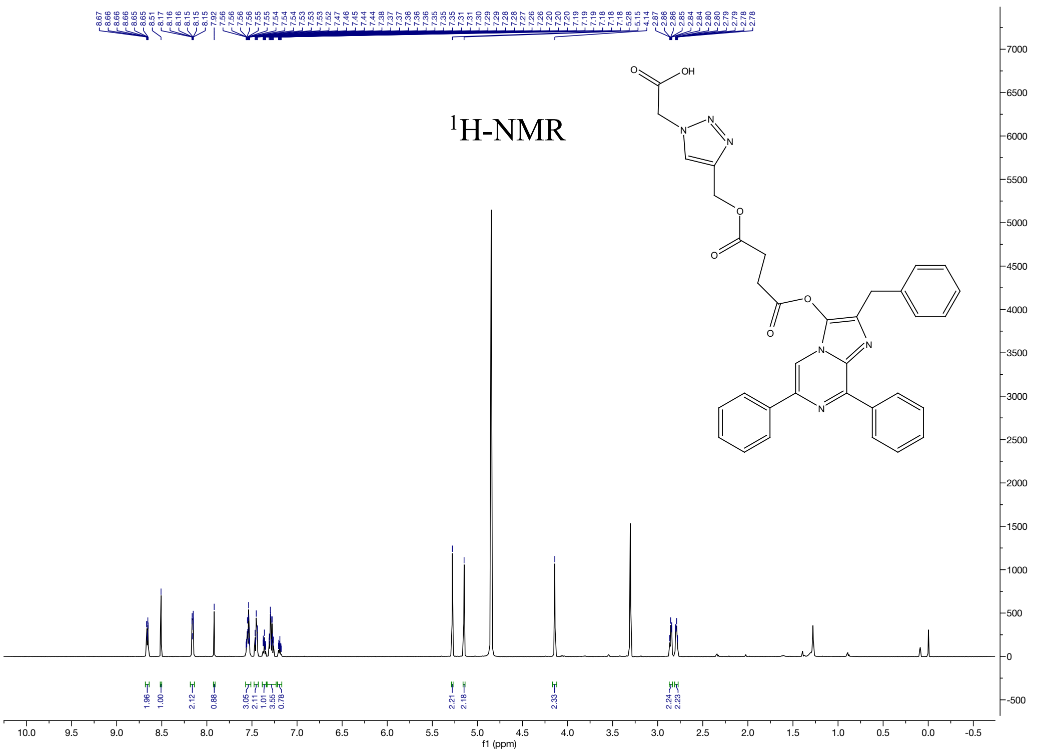

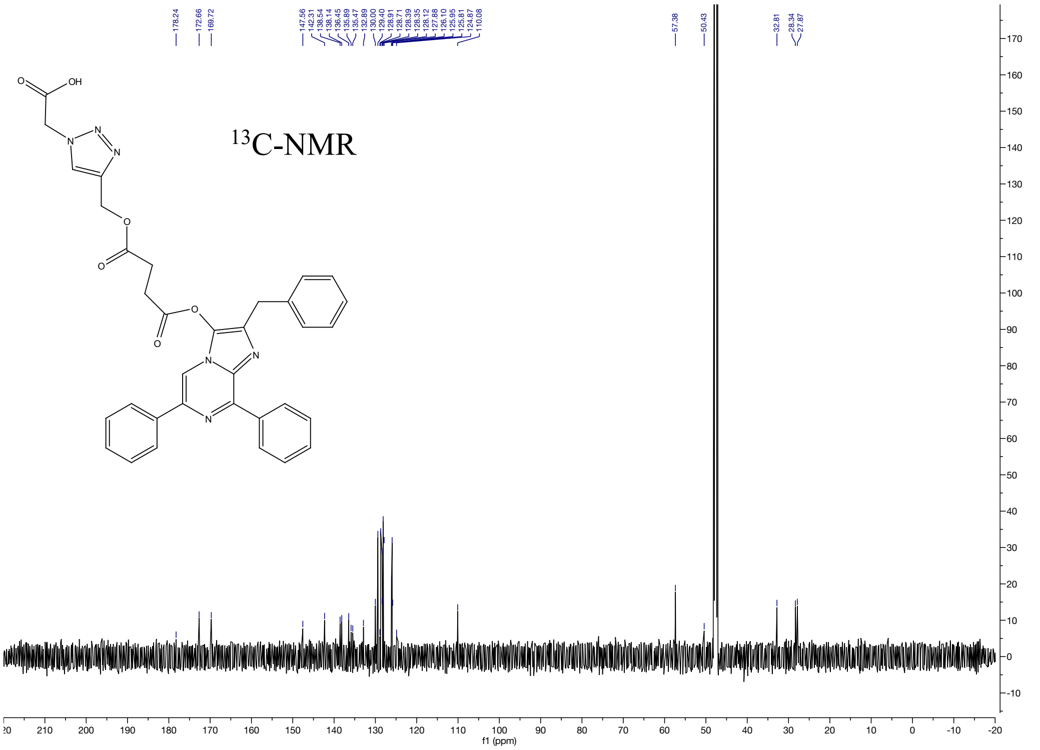

Supplementary Fig 16. NMR spectra for the indicated compound.

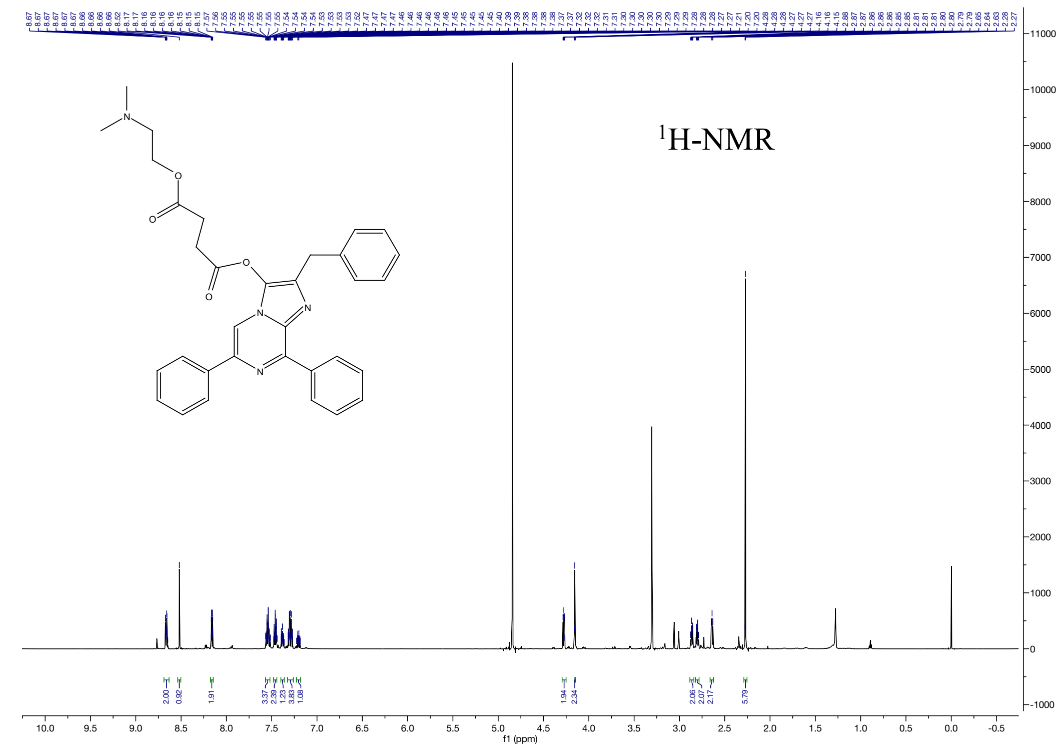

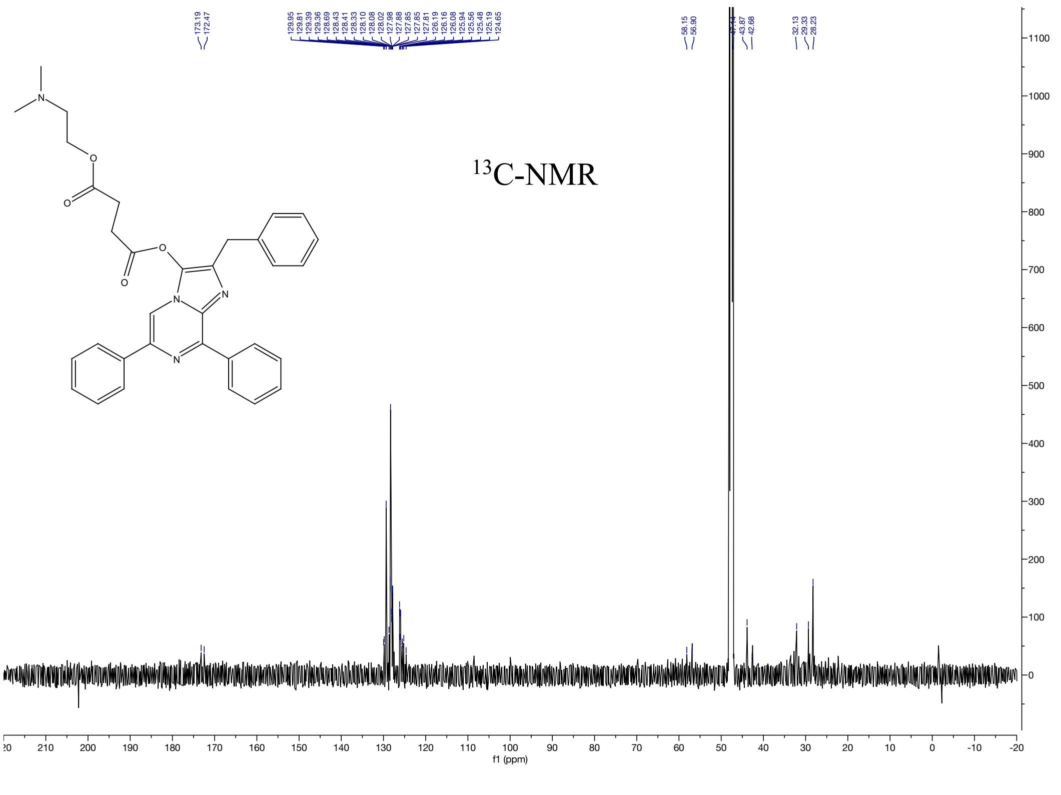

Supplementary Fig 17. NMR spectra for the indicated compound.

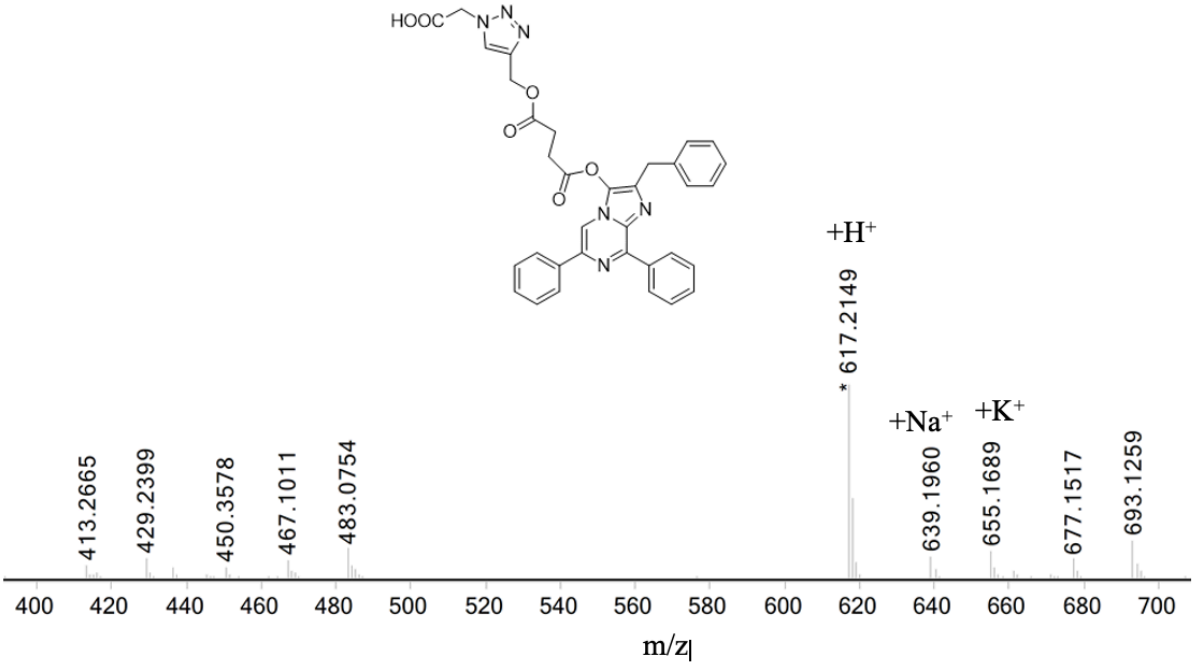

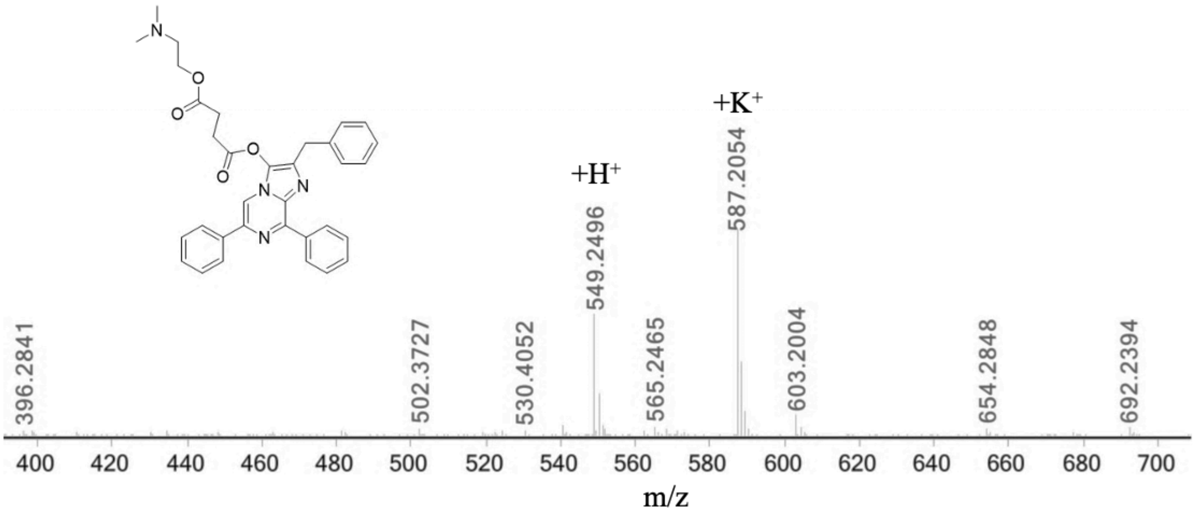

Supplementary Fig 18. HR-MS spectra for the indicated compounds.

Supplementary Table 1. Oligonucleotides used in this work

Oligo name	Sequence (5′→3′)
pBAD_FW_BREP1_NNK1	CAGTACGAACGCTCCGAGGGCCGCNNKGAAGATTTCGTTGGGGAC
pBAD_RV_BREP1_NNK1	GTCCCCAACGAAATCTTCMNNGCGGCCCTCGGAGCGTTCGTACTG
pBAD_FW_BREP1_NNK2	CAGTACGAACGCTCCGAGGGCCGCNNKACACTCGAAGATTTCGTTGGGGAC
pBAD_RV_BREP1_NNK2	GTCCCCAACGAAATCTTCGAGTGT MNNGCGGCCCTCGGAGCGTTCGTACTG
pBAD_FW_BREP_3NDT	CAGTACGAACGCTCCGAGGGCCGCNDTNDTNDTACACTCGAAGATTTCGTTGGGGAC
pBAD_RV_BREP_3NDT	GTCCCCAACGAAATCTTCGAGTGTAHNAHNAHNGCGGCCCTCGGAGCGTTCGTACTG
pBad_FW1_mScarlet	GACGATAAGGATCCGAGCTCGAGCATGGTGAGCAAGGGCGAG
pBad_RV1_teLuc(133)	CTTCTGTCAGTTGGTCATGCATAATCAGGGTCCCTGTTACAGTG
pBad_FW2_CaM	CACTGTAACAGGGACCCTGATTATGCATGACCAACTGACAGAAG
pBad_RV2_M13	GATAATTTTGTTGCCTTTCCAGAGCTCCAGTGCCCCGGAGCTGGAGA
pBad_FW3_teLuc(134)	TCTCCAGCTCCGGGGCACTGGAGCTCTGGAAAGGCAACAAAATTATC
pBad_RV3_teLuc(168)	TCTCATCCGCCAAAACAGCCAAGCTTTTACGCCAGAATGCGTTCATG
pBad_FW_EP	ATGACGATAAGGATCCGAGCTCGAG
pBad_RV_EP	CTCATCCGCCAAAACAGCCAAGCTTTTA
pBad_FW_BRIC	CCGCTCGAGCATGGTGAGCAAGGGCGAGG
pBad_RV_strep(BRIC)	CCCAAGCTTTTATTTTTCGAACTGCGGGTGGCTCCACGCCAGAATGCGTTCATGC
pBad_FW_His(CaMBI)	ATAAGGATCCGAGCTCGAGCATGGTGAGCAAGGGCGAG
pBad_RV_strep(CaMBI)	TTTTTCGAACTGCGGGTGGCTCCACTTATAGAGTTCATCCATTC
pBad_RV_ext	TCATCCGCCAAAACAGCCAAGCTTTTATTTTTCGAACTGCGGGTGGCTCCAC
pcDNA3_FW_HindIII	TACGACTCACTATAGGGAGACCCAAGCTTGCCACCATGGTGAGCAAGGGCGAGGCAG
pcDNA3_RV_XhoI	TAGGGCCCTCTAGATGCATGCTCGAGTTACGCCAGAATGCGTTCATGCAGAC
pAAV_hSyn_FW(BRIC/BREP)	ATTCAAGCTGCTAGCAAGGATCCCGCCACCATGGTGAGCAAGGGCGAG
pAAV_hSyn_RV(BRIC/BREP)	TCCAGAGGTTGATTATCGATAAGCTTTTACGCCAGAATGCGTTCATGCAGAC
pAAV_hSyn_FW(CaMBI)	ATTCAAGCTGCTAGCAAGGATCCCGCCACCATGGTGAGCAAGGGCGAG
pAAV_hSyn_RV(CaMBI)	TCCAGAGGTTGATTATCGATAAGCTTTTACTTATAGAGTTCATCCATTC
