# Supplementary material for: A luciferase prosubstrate and a red bioluminescent calcium indicator for imaging neuronal activity in mice
Source: Nat Commun. 2022 Jul 8;13:3967. doi: 10.1038/s41467-022-31673-x (PMC9270435; doi:10.1038/s41467-022-31673-x)
Supplement: Supplementary file 2 — Reporting Summary [file 41467_2022_31673_MOESM2_ESM.pdf]

nature portfolio

Corresponding author(s): Hui-wang Ai

Last updated by author(s): Jun 24, 2022

Reporting Summary

Nature Portfolio wishes to improve the reproducibility of the work that we publish. This form provides structure for consistency and transparency in reporting. For further information on Nature Portfolio policies, see our Editorial Policies and the Editorial Policy Checklist.

Statistics

For all statistical analyses, confirm that the following items are present in the figure legend, table legend, main text, or Methods section.

n/a	Confirmed	
☐	☒	The exact sample size (*n*) for each experimental group/condition, given as a discrete number and unit of measurement
☐	☒	A statement on whether measurements were taken from distinct samples or whether the same sample was measured repeatedly
☐	☒	The statistical test(s) used AND whether they are one- or two-sided<br>*Only common tests should be described solely by name; describe more complex techniques in the Methods section.*
☒	☐	A description of all covariates tested
☐	☒	A description of any assumptions or corrections, such as tests of normality and adjustment for multiple comparisons
☐	☒	A full description of the statistical parameters including central tendency (e.g. means) or other basic estimates (e.g. regression coefficient) AND variation (e.g. standard deviation) or associated estimates of uncertainty (e.g. confidence intervals)
☐	☒	For null hypothesis testing, the test statistic (e.g. *F*, *t*, *r*) with confidence intervals, effect sizes, degrees of freedom and *P* value noted<br>*Give P values as exact values whenever suitable.*
☒	☐	For Bayesian analysis, information on the choice of priors and Markov chain Monte Carlo settings
☒	☐	For hierarchical and complex designs, identification of the appropriate level for tests and full reporting of outcomes
☒	☐	Estimates of effect sizes (e.g. Cohen's *d*, Pearson's *r*), indicating how they were calculated

Our web collection on statistics for biologists contains articles on many of the points above.

Software and code

Policy information about availability of computer code

Data collection	Bruker TopSpin (Version 3.5pl4) was used to control Bruker Avance III 600 for NMR spectrum collection. MassLynx (Version 4.2) was used to control Waters Prep 150 -SQD2 LC-MS. Agilent MassHunter Workstation Data Acquisition (Version B.09) was used to acquire HR-MS on an Agilent 6545 Q-TOF LC/MS system via direct infusion. BMG Labtech CLARIOstar Plus Reader Software (Version 5.70 R2) was used to control the plate reader for bioluminescence measurements. Leica LAS X (Version 3.5.7) or MicroManager (Version 2.0) was used to acquire microscopic images. UVP VisionWorksLS software (Version 8.6) and MicroManager (Version 2) were used for darkbox imaging.
Data analysis	MestReNova (Version 12.0.3) was used to analyze NMR data. MassLynx (Version 4.2) or Agilent MassHunter Workstation Quantitative Analysis Navigator (Version B.08) was used to analyze MS data. BMG Labtech CLARIOstar MARS Data Analysis Software (Version 3. 42 R5) was used to analyze results from the CLARIOstar Plus Plate Reader. Fiji (ImageJ Version 2.1) was used to analyze microscopic images. Microsoft Excel (Version 15.21.1), GraphPad Prism (Version 8), and Affinity Designer (Version 1.10.4) were used to analyze data and prepare figures for publication.

For manuscripts utilizing custom algorithms or software that are central to the research but not yet described in published literature, software must be made available to editors and reviewers. We strongly encourage code deposition in a community repository (e.g. GitHub). See the Nature Portfolio guidelines for submitting code & software for further information.

Data

Policy information about availability of data

All manuscripts must include a data availability statement. This statement should provide the following information, where applicable:

- Accession codes, unique identifiers, or web links for publicly available datasets
- A description of any restrictions on data availability
- For clinical datasets or third party data, please ensure that the statement adheres to our policy

The plasmids pcDNA3-BREP (#172337), pcDNA3-BRIC (#172338), pAAV-hSyn-BREP (#172340), pAAV-hSyn-BRIC (#172341), and pBAD-BRIC (#172343) and their sequence information have been deposited to Addgene. All key data and experimental methods are presented in the main text or the supplementary materials. Other biological materials are available from the corresponding author upon request. Source data are provided with this paper. Protein structures (Entries 2BBM, 7MJB, and 5LK4) used for creating graphs can be accessed from the RSCB Protein Data Bank.

Field-specific reporting

Please select the one below that is the best fit for your research. If you are not sure, read the appropriate sections before making your selection.

☒ Life sciences ☐ Behavioural & social sciences ☐ Ecological, evolutionary & environmental sciences

For a reference copy of the document with all sections, see nature.com/documents/nr-reporting-summary-flat.pdf

Life sciences study design

All studies must disclose on these points even when the disclosure is negative.

Sample size	No statistical methods were used to predetermine sample size. For in vitro and cellular experiments, sample sizes are usually 3 or more so that mean and standard deviation or standard error can be calculated. For in vivo experiments, sample sizes were determined based on similar studies in our own lab or reported by other researchers. Sample size and the number of replications for each experiments are presented in relevant figure legends.
Data exclusions	No data exclusions were performed.
Replication	Replication information for all data reported in this study is stated in figure legends. Experimental findings were reliably reproduced.
Randomization	Cell cultures and mice were randomly assigned to treatment groups.
Blinding	Blinding was not implemented during sensor development and in vitro and cellular characterization experiments. For in vivo footshock and KA-induced seizure experiments, the investigator who acquired and analyzed data was blinded to the group allocation during experiments.

Reporting for specific materials, systems and methods

We require information from authors about some types of materials, experimental systems and methods used in many studies. Here, indicate whether each material, system or method listed is relevant to your study. If you are not sure if a list item applies to your research, read the appropriate section before selecting a response.

Materials & experimental systems

n/a	Involved in the study	
☒	☐	Antibodies
☐	☒	Eukaryotic cell lines
☒	☐	Palaeontology and archaeology
☐	☒	Animals and other organisms
☒	☐	Human research participants
☒	☐	Clinical data
☒	☐	Dual use research of concern

Methods

n/a	Involved in the study	
☒	☐	ChIP-seq
☒	☐	Flow cytometry
☒	☐	MRI-based neuroimaging

Eukaryotic cell lines

Policy information about cell lines

Cell line source(s)	HEK 293T and HeLa cells (ATCC)
Authentication	Not authenticated in our lab, but HEK 293T and HeLa cells were directly purchased from ATCC (authenticated using short tandem repeat per the vendor).
Mycoplasma contamination	HEK 293T and HeLa cells were routinely tested for mycoplasma contamination every 2-3 months using qPCR, and the test results were negative.
Commonly misidentified lines (See ICLAC register)	No commonly misidentified line was used in this study.

Animals and other organisms

Policy information about studies involving animals; ARRIVE guidelines recommended for reporting animal research

Laboratory animals	BALB/cJ mice (The Jackson Laboratory, #000651, 8 weeks of age) and C57BL/6J mice (The Jackson Laboratory, #000664, 8 weeks of age) of both sex were used. All mice were hosted in a temperature-controlled room (~23 °C) with a 12 h/12 h dark-light cycle and ~50% humidity.
Wild animals	The study did not involve wild animals.
Field-collected samples	The study did not involve field-collected samples.
Ethics oversight	All animal experiments were conducted according to the approval and guidelines of the University of Virginia Institutional Animal Care and Use Committee.

Note that full information on the approval of the study protocol must also be provided in the manuscript.
