# Supplementary material for: A luciferase prosubstrate and a red bioluminescent calcium indicator for imaging neuronal activity in mice
Source: Nat Commun. 2022 Jul 8;13:3967. doi: 10.1038/s41467-022-31673-x (PMC9270435; doi:10.1038/s41467-022-31673-x)
Supplement: Supplementary file 3 — Description of Additional Supplementary Files [file 41467_2022_31673_MOESM3_ESM.pdf]

Title: Supplementary Movie 1.
Description: BLI of BRIC-expressing HeLa cells in response to histamine.

Title: Supplementary Movie 2.
Description: BLI of BRIC-expressing mouse neurons in response to high-K^+ depolarization.

Title: Supplementary Movie 3.
Description: BLI of a BRIC-expressing hippocampal brain slice in response to high-K^+ depolarization.

Title: Supplementary Movie 4.
Description: BLI of a live mouse with BRIC expressed in the BLA in response to 13 repeats of footshock stimulation.

Title: Supplementary Movie 5.
Description: BLI of a live mouse with BRIC expressed in the hippocampus during KA-induced seizures.
